# Conceptual DFT as a chemoinformatics tool for the study of the Taltobulin anticancer peptide

**DOI:** 10.1186/s13104-019-4478-7

**Published:** 2019-07-19

**Authors:** Norma Flores-Holguín, Juan Frau, Daniel Glossman-Mitnik

**Affiliations:** 1 Laboratorio Virtual NANOCOSMOS, Departamento de Medio Ambiente y Energía, Centro de Investigación en Materiales Avanzados, Miguel de Cervantes 120, Complejo Industrial Chihuahua, 31136 Chihuahua, Mexico; 2 Departament de Química, Universitat de les Illes Balears, 07122 Palma, Spain

**Keywords:** Taltobulin, Conceptual DFT, Bioactivity scores

## Abstract

**Objective:**

A well-behaved model chemistry previously validated for the study of the chemical reactivity of peptides was considered for the calculation of the molecular properties and structure of the Taltobulin anticancer peptide. A methodology based on Conceptual Density Functional Theory (CDFT) was chosen for the determination of the reactivity descriptors.

**Results:**

The molecular active sites were associated with the active regions of the molecule were associated with the nucleophilic and electrophilic Fukui functions. Finally, the bioactivity scores for the Taltobulin peptide are predicted through a homology methodology relating them with the calculated reactivity descriptors.

## Introduction

The biodiversity of the marine environment and the associated chemical diversity constitute a practically unlimited resource of new antitumor agents in the field of the development of marine bioactive substances [1].

Hemiasterlins comprise a small family of naturally occurring *N*-methylated tripeptides with highly alkylated unnatural amino acids, was originally isolated from the sponge Hemiasterella minor (class Demospongiae, order Hadromedidia, family Hemiasterllidae). Hemiasterlins act as potent tumor growth inhibitors [2, 3].

A synthetic analogue of hemiasterlin, taltobulin (HTI-286) has been synthesized in which the 3-substituted indole ring was replaced by phenyl group. Taltobulin inhibits the polymerization of purified tubulin and disrupts microtubule organization in cells. Then, it is considered as a potent inhibitor of proliferation and has substantially less interaction with multidrug resistance protein than currently used antimicrotubule agents [4].

Assuming that an understanding of the chemical interactions is essential for the development of new pharmaceutical drugs, in this work we will be studying the chemical reactivity properties of Talbodulin by resorting to the Conceptual DFT methodology, which will allow the determination of the global properties as well as the local properties for the prediction of the active reaction sites, both electrophilic and nucleophilic. In a similar way, the descriptors of bioactivity (bioactivity scores) will be established through a procedure described in the literature [5, 6] trying to relate them with the global and local CDFT reactivity descriptors that result from a calculation protocol based on DFT already validated by our group in previous research [7–14].

## Main text

### Computational methodology

The ChemAxon Calculator Plugins for Conformers Searching available in Marvin View 17.15.0 were used to generate 3D structures from SMILES strings, and to propose low energy conformers for structure property prediction and calculation. For the molecule considered in the current study, the lowest energy conformers were used as a starting point for the geometry optimization. The geometries of all the selected conformers were optimized with the DFTBA program. The molecular structures of the five lowest energy conformers were reoptimized by resorting to the MN12SX/Def2TZVP/H2O model chemistry. The optimized structures were confirmed to be real minima by vibrational frequency analysis (no imaginary frequency).

### Results and discussion

**Fig. 1:**
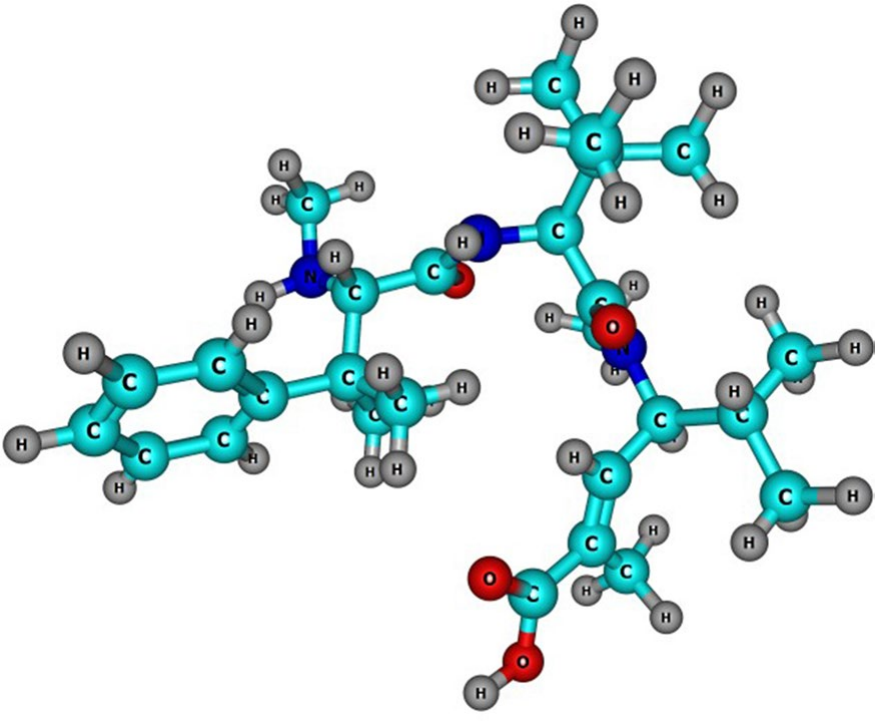
Graphical sketch of the molecular structure of the Taltobulin peptide

The molecular structures of the optimized conformers of Taltobulin obtained as mentioned in the previous section, and whose graphical sketch is shown in Fig. 1, has been submitted to optimization in absence of solvent by resorting the DFTBA model available in Gaussian 09 [15] and then reoptimized using the MN12SX/Def2TZVP/H2O model chemistry mentioned in that section. Having verified that each of the structures corresponded to the minimum energy conformations by running a frequency calculation analysis, the electronic properties were determined by using the same model chemistry.

Becke has recently mentioned that the adiabatic connection and the ideas of Hohenberg, Kohn, and Sham applying only to electronic ground states is a common misconception [16]. In this regard, the HOMO–LUMO gap within the KS model represents a nice estimation of the lowest excitation energy [17]. Under this assumption, the determination of the maximum wavelength absorption of the Taltobulin peptide was performed by conducting a ground-state calculation with the aforementioned density functional at the same level of model chemistry and theory to obtain the HOMO–LUMO gap and subsequently, the $\lambda _{max}$. The electronic energy of the neutral molecular system of Taltobulin, the HOMO and LUMO orbital energies, and the maximum absorption wavelengths $\lambda _{max}$ calculated with the MN12SX/Def2TZVP/H2O model chemistry are − 1517.422 au, − 6.240 eV, − 1.733 eV, and 275 nm, respectively.

According to the results obtained when studying melanoidins [7–13] as well as peptides from marine sources [14], it can be said that the calculations performed with the MN12SX density functional render HOMO and LUMO energies that satisfy the approximate Koopmans’ theorem. Thus, the application of the KID procedure will be justified. The global reactivity descriptors Electronegativity $\chi$ [18, 19], Global Hardness $\eta$ [18, 19], Electrophilicity $\omega$ [20], Electrodonating Power $\omega ^{-}$ [21], Electroaccepting Power $\omega ^{+}$ [21] and Net Electrophilicity $\Delta \omega ^{\pm }$ [22] were calculated by resorting to the HOMO and LUMO energies determined with the MN12SX density functional with results being $\chi$ = 3.986 eV, $\eta$ = 4.507 eV, $\omega$ = 1.763 eV, $\omega ^{-}$ = 5.800 eV, $\omega ^{+}$ = 1.814, and $\Delta \omega ^{\pm }$ = 7.614 eV. The interested reader in the mathematical formulations of these reactivity descriptors is referred to the original works and to our previous research on the field [7–14]. As expected from the molecular structure of this species, its electrodonating ability is more important that its electroaccepting character.

**Fig. 2:**
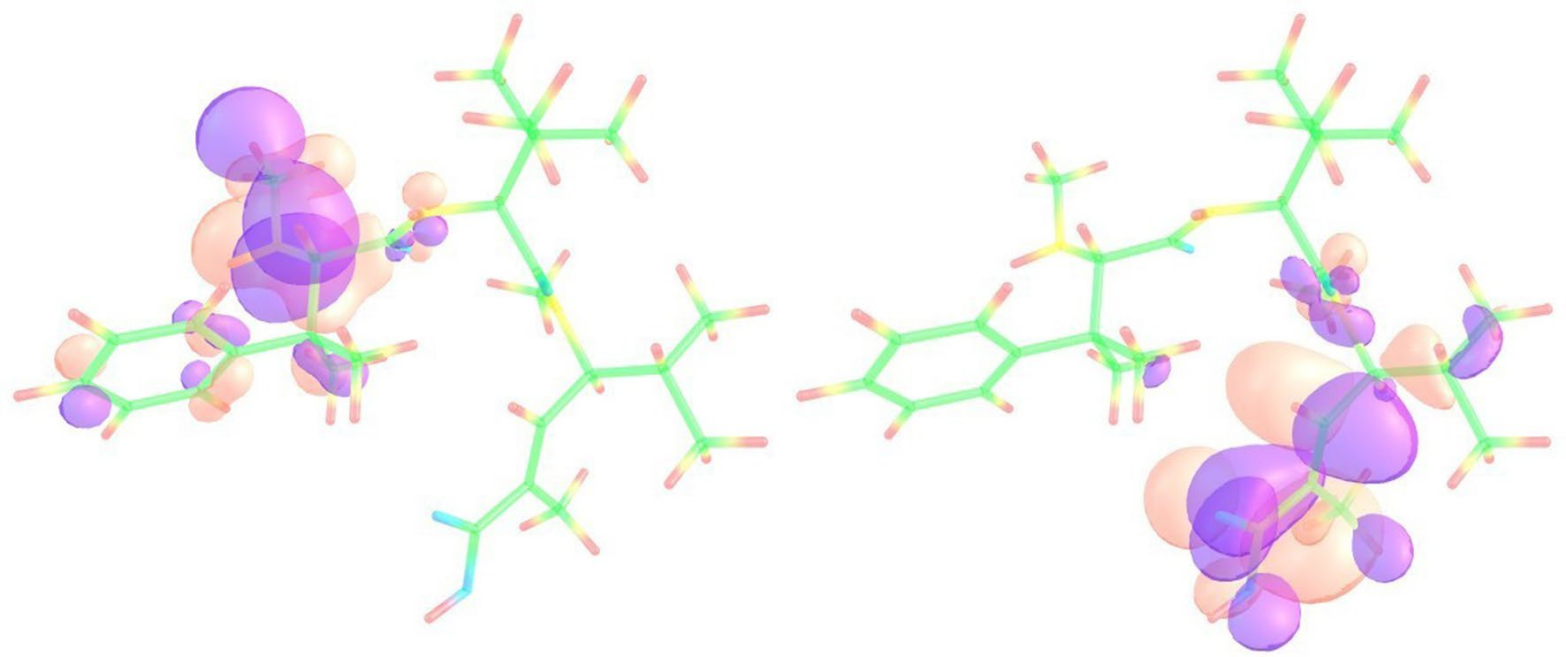
Graphical representation of the Electrophilic Fukui function $f^{-}(\mathbf {r})$ (left) and Nucleophilic Fukui functions $f^{+}(\mathbf {r})$ (right) of the Taltobulin peptide

We now turn our attention to the local descriptors of chemical reactivity, namely the Electrophilic Fukui function $f^{-}(\mathbf {r})$ [18, 19, 23], the Nucleophilic Fukui function $f^{+}(\mathbf {r})$ [18, 19, 23] and the Dual Descriptor (DD) ${\Delta }f(\mathbf {r})$ [24–28]. As for the case of the global reactivity descriptors, the interested reader in the mathematical formulations of these reactivity descriptors is referred to the original works and to our previous research on the field [7–14]. The Electrophilic Fukui functions $f^{-}(\mathbf {r})$ and Nucleophilic Fukui functions $f^{+}(\mathbf {r})$ for the Taltobulin peptide are shown in Fig. 2.

Within the field of Chemoinformatics applied to the discovery of new pharmaceutical drugs, it is usual to verify the drug-likeness of the involved molecules resorting to some empirical rules, as for example, the Lipinski Rule of Five (Ro5) [29]. This can be achieved by using the readily available Molinspiration software and the results for the case of Taltobulin are presented next as miLog*P* (the octanol/water partition coefficient) = 4.43, TPSA (the molecular polar surface area) = 98.73, nAtoms (the number of atoms of the molecule) = 34, nON (the number of hydrogen bond acceptors) = 7, nOHNH (the number of hydrogen bond donors) = 3, nviol (the number of violations of the Ro5) = 0, nrotb (the number of rotatable bonds) = 11, volume (the molecular volume) = 479.94 and MW (the molecular weight) = 473.66. Although this criteria cannot always be applied in general to peptides, it can be seen from the previous results that for Taltobulin the number of violations of the Ro5 is 0, which means that Taltobulin can be considered as a druggable molecule.

As the next step, a new task was accomplished by resorting to the same software for the determination of the bioactivity scores for different drug targets whose values for the Taltobulin peptide are GPCR Ligand = 0.43, Ion Channel Modulator = 0.15, Kinase Inhibitor = - 0.12, Nuclear Receptor Ligand = 0.19, Protease Inhibitor = 0.68 and Enzyme Inhibitor = 0.42.

These bioactivity scores for organic molecules can be interpreted as active (when the bioactivity score is > 0), moderately active (when the bioactivity score lies between − 5.0 and 0.0) and inactive (when the bioactivity score $<-$5.0). That means that the Taltobulin peptide can be considered a potentially bioactive as a Protease inhibitor, besides being able to act a ligand for GPCR and as an Enzyme inhibitor.

## Limitations

In this research note, we have presented the results of a study of the chemical reactivity of a the Taltobulin anticancer peptide on the basis of the Conceptual DFT as a tool to explain the molecular interactions, the molecular properties related to bioavailability and the bioactivity scores.

However, this information is insufficient to be considered for peptidomimetics studies and additional results from other peptides will be necessary.

## Abbreviations

DFT: Density Functional Theory
CDFT: Conceptual Density Functional Theory
SMILES: Simplified Molecular Input Line Entry Specification
DFTBA: Density Functional Tight Binding Model A
HOMO: Higher Occupied Molecular Orbital
LUMO: Lower Unoccupied Molecular Orbital
KS: Kohn–Sham
KID: Koopmans in DFT
DD: Dual Descriptor
Ro5: Lipinski Rule of Five
GPCR: G Protein-coupled Receptor
